# Non-Face to Face Student Learning Time: An ocean in Medical Education

**DOI:** 10.12669/pjms.35.3.935

**Published:** 2019

**Authors:** Ashfaq Akram

**Affiliations:** *Dr. Ashfaq Akram, Curriculum Specialist, Medical Education Department, College of Medicine, King Saud University, Riyadh, Saudi Arabia. Email: ashfaqakram@hotmail.com*

Student learning time (SLT), based on its characteristics, is classified as Face to Face (F2F) and Non-Face to Face (NF2F). The former, conducted *in the classroom, is* measurable in time units where information is delivered in a quantified time period by a teacher while latter, learning is done *outside the classroom* in the absence of instructor and its time limit is still hypothesized. We could say it is a bipolar educational setting and partly enfolded domain in medical education. For example, a lecture is 60 minutes of organized learning activity in the classroom is delivered by an instructor to the students.^1^ If there are 30 lectures in a particular course or module, F2F students’ learning would be 30 hours. The time spent by students to study those lecture topics, in apple pie order to reproduce the contents as targeted or expressed in the learning outcomes or objectives of the course outside the classroom, is NF2F. Indeed, we exactly don’t know NF2F; therefore, we call it an ‘Ocean of NF2F’.

The significance of SLT is inevitable because it contributes directly to academic student load, which is widely based on the Carnegie Unit system, calculated in most U.S. medical schools in credit hours.^2^ Considering lecture, one hour systemized learning activity of students delivered by a tutor or instructor is a basic teaching method and assembles credit hour. Its unit represents a single subject taught for one classroom period for five days a week (USNEI 2008). In other words, credit, other than the academic heap, is a change in knowledge, skill, and attitude.^3^ Hence, it requires F2F and NF2F phases of learning. To achieve a credit, F2F and NF2F both are calculated in addition to the assessment method. However, this is a biphasic mirror of one side crystal clear surface made with definitely calculated time slots while the other side is vague.

Currently, medical educationists estimate the academic load saying, for one F2F hour (contact hour), 2 hours (NF2F) would be needed.^4^ Keeping this allegory, cerebral aneurysm, Meningitis, and Encephalitis delivered each as a lecture of one hour would require two hours for all students. The question arises, ‘Do we know two hours is the real-time taken by ALL medical students? Not yet. Considering other aspects, humans have different IQs. Therefore, equal NF2F time is not possible. Regardless of IQ tests which only measure the intelligence and experts suggest that not only other important elements including social and emotional factors contribute to intelligence but also actually matter more than IQ when it comes to determining success in life. But IQ doesn’t correlate the success in the medical profession.^5^

Attending classes regularly, continuous studying with dedication, and practicing skills to achieve success in the examination, is the factors that ensure mostly the best medical students could stand. Following this modish, other than lecture, Simulation teaching, Bedside teaching; Problem based learning, Seminar, Tutorial, etc., are also well known F2F teaching modalities with fixed time slots in all medical schools, each one last for either one or two hours as F2F session regardless of teacher or student center. The chosen topics for these teaching practices are awaiting the educationists to estimate the NF2F learning time.

Presently, in most medical schools, basic medical sciences are integrated with clinical sciences and concepts are taught and learned in the classroom. Despite integration, even the traditional curriculum, the phases of SLT are the same. We take another example of ‘Central nervous system. The anatomy and physiology of Hypothalamus cannot be as easy as normal physiology of a human cell. But both are delivered a lecture of 60 minutes. The Kreb cycle of glucose would need more time as compared to the classification of carbohydrate. Similarly, pharmacokinetics cannot be equal in understanding chemotherapy drugs. An exemplary figure is given below.

**Figure:**
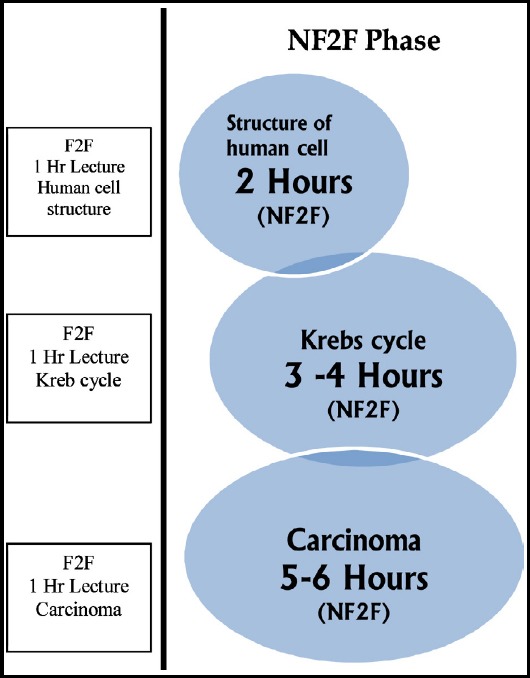
Two phases of students’ learning F2F and NF2F.

NF2F is an ocean that has yet to be discovered not only for medical science but also for other natural sciences. This ocean is on the students’ side so the factors associated with students’ physical and mental personality are strongly expected to affect its measurement.

Ironically; the NF2F for Psychomotor learning is more complex and needs much scrutiny and heed of medical educationists. For example, there would be a significant difference in terms of time needed to learn and master in psychomotor skills of auscultation of heart, taking a complete history of a cardiac patient, traumatic brain injury or removing an impacted 3^rd^ molar tooth. There are significant time variations in learning neuronal skills and respiratory skills. The evidence is lacking, as yet, therefore it is speculated students may utilize 8-12 hours to perform a certain skill in a good or acceptable manner.

Inspired by innovation in developments of curriculum models that emphasize the update knowledge, the community context of disease, students attitude and behaviour, teamwork, developing critical thing and leadership qualities, we discern a need to individualize the NF2F while also promoting teaching and probing assessment methods, improving the self-students learning, patients care environment to eliminate the hazy and blurred picture of credit hours, reserved time slots. NF2F time would deviate for different topics and chapters for students. Apparent and outward benefits would include more reliability in credit hour calculation, varied time slots for different topics, re-weight of different disciplines, and re-allocating the teaching methods. In sum up, it would bring a revolutionary change in curriculum model and educational settings.
